# From existence checking to semantic auditing: citation verifiability in the age of AI

**DOI:** 10.3389/frma.2026.1927189

**Published:** 2026-09-10

**Authors:** Libor Ansorge

**Affiliations:** T. G. Masaryk Water Research Institute, Podbabská, Prague, Czechia

**Keywords:** agentic AI, artificial intelligence (AI), citation classification, citation inflation, citation ontology, citations, fake citations, semantic auditing

## Abstract

This paper reflects on the current crisis of academic integrity caused by the phenomenon of fabricated and semantically incorrect citations. In the era of ‘fast science' and permanent citation inflation, human reviewers face extreme cognitive overload and time pressure, making it impossible to consistently verify literature sources. As a key solution, this paper proposes the deployment of autonomous AI agents (Agentic AI) designed for the deep semantic auditing of manuscripts. This technological shift is complemented by an innovative proposal to introduce mandatory authorial ‘citation reports' that operationalize the concepts of citation classification. The resulting synergy between human expertise and machine precision relieves reviewers of their mechanical burden and can help strengthen the rigor, transparency, and credibility of peer review.

## Citation-verifiability problems

Academic research is inherently a cumulative process, building on the foundations laid by our predecessors. In this complex ecosystem, citing sources represents the cornerstone of academic integrity. It is not a mere formal or bureaucratic obligation, but a fundamental ethical pillar of scholarly work. Accurate citation serves two key roles: on the one hand, it guarantees due credit to the authors of original ideas and groundbreaking works; on the other, it ensures transparency and verifiability of the claims made (Merton, 1968). Without a reliable and accurate citation apparatus, the line between authentic scientific contribution and intellectual dishonesty becomes blurred, directly threatening the trust of the professional community and the public in science as a whole.

In today's digital and highly competitive environment, however, the scientific community is facing a new and deeply disturbing phenomenon: fake citations, which refer to scientific works that do not actually exist. This serious offense against academic ethics has its roots in both the structural problems of modern science and rapid technological development. The first major factor is the relentless pressure on publication quantity, known as the “publish or perish” principle, which has given rise to commercial “paper mills.” These dubious entities have been generating fictitious texts on an industrial scale since the 1960s (Dickerson, 2007), often with a completely fabricated citation apparatus, in order to artificially improve the metrics of paying clients. The second, no less risky source is the uncritical use of generative artificial intelligence. Large language models (LLMs) without advanced verification tools have an inherent tendency toward so-called hallucinations—in the interest of text fluency, they can generate plausible-sounding author names, study titles, and non-existent Digital Object Identifiers (DOIs) with complete conviction (Walters and Wilder, 2023; Bhattacharyya et al., 2023). The infiltration of the scientific space with these “non-existent” sources directly threatens the ability of science to accumulate genuine knowledge, as it creates the illusion of a scientific consensus built on a mere vacuum.

While completely fabricated references represent deliberate fraud, an equally serious and much more widespread problem in academic practice—historically well-documented (Lacey et al., 1985)—is the mis-citation of real works. These offenses against scientific writing ethics often stem from two primary causes. The first is the author's indifference and superficiality, where the author has not read the cited work at all and has mechanically copied the reference from secondary literature. This practice leads to gross misinterpretations of original ideas that circulate and become entrenched within the scientific community for years without verification (Rekdal, 2014). The second cause is the authors' attempt to use citations as mere ‘filler' ballast to artificially boost their paper's prestige. They operate under the delusion that a longer, more robust list of references makes their text appear more erudite to editors and reviewers. The result is dozens of references that have no semantic relevance to the actual content of the cited works. Both of these practices fundamentally distort scientific discourse: they transform the citation apparatus from a tool for rigorous knowledge verification into a mere decoration, creating informational noise that paralyzes standard critical evaluation.

Furthermore, as highlighted in recent discussions on academic standards, these citation deficiencies are often compounded by a systemic lack of literature due diligence, where authors fail to conduct thorough literature searches prior to manuscript submission. While semantic web initiatives and frameworks—such as the PORTAL-DOORS Project and associated FAIR metrics (Craig and Taswell, 2025)—have proposed formal metric-based approaches to identify reference omissions, miscitations, and ghosting within digital repositories, the practical enforcement of these standards during active peer review remains a major operational challenge.

Moreover, the demand for honesty and accuracy in referencing is directly challenged by a deeper structural transformation in scholarly publishing: the exponential increase in the number of citations per article over time. This phenomenon, increasingly referred to in quantitative studies of science as “citation inflation,” fundamentally densifies the connections within the global scientific network (Lin et al., 2023; Petersen et al., 2024). While in past decades, printed journals strictly limited the scope of manuscripts and the number of permitted references due to the limited physical capacity of paper pages, the digital era and the rise of online “megajournals” have completely erased these material restrictions. Coupled with the explosive growth in the total number of published texts and the easy online availability of bibliometric databases and citation tools, the average length of reference lists has steadily increased across all scientific disciplines over the past decade (Dai et al., 2021). This trend is further amplified by systematic pressure on metrics such as the h-index or global university rankings, which motivates authors to overuse references without deeper semantic justification.

## Limitations of current review practice

For a human reviewer, this citation inflation represents an unsustainable burden: in an environment where a single article commonly cites fifty or more works, manually cross-checking the semantic correctness of each citation becomes practically infeasible for an individual. This burden on reviewers is further exacerbated by the increasing pressure to keep review times short. In the current era of so-called “fast science,” authors logically expect prompt publication of their findings (Powell, 2013) to secure academic priority or meet grant deadlines. Publishers respond to these expectations by shortening review deadlines—often to a mere 10 days or 2 weeks. Reviewers, who perform this highly responsible activity voluntarily and without compensation, thus find themselves under immense time constraints. They are required to conduct a rigorous scientific audit of a complex text, yet under conditions that practically mandate superficial reading. In a system structured in this manner, the thorough verification of the citation apparatus and tracing of original sources are the first elements to be sacrificed for purely pragmatic, time-saving reasons.

As a reviewer, I primarily focus on whether authors cite the real originators of ideas or whether they conveniently rely on secondary sources. I also monitor whether information that is clearly not the result of the authors' own work is properly cited (Ansorge and Stejskalová, 2023)—specifically, whether there is premature invocation of the concept of “Obliteration by Incorporation,” where external contributions are prematurely treated as generally known, uncited facts (Merton, 1968; Garfield, 1975). In suspicious instances, I verify whether the cited source actually supports the claim and critically assess the relevance of the references, particularly to detect intentional self-citation. However, as detailed in the previous paragraph, conducting such a comprehensive cognitive audit for each individual reference is practically infeasible for a human evaluator under the conditions of current citation inflation and restrictive review deadlines. Consequently, the majority of citations remain unchecked.

**Figure 1:**
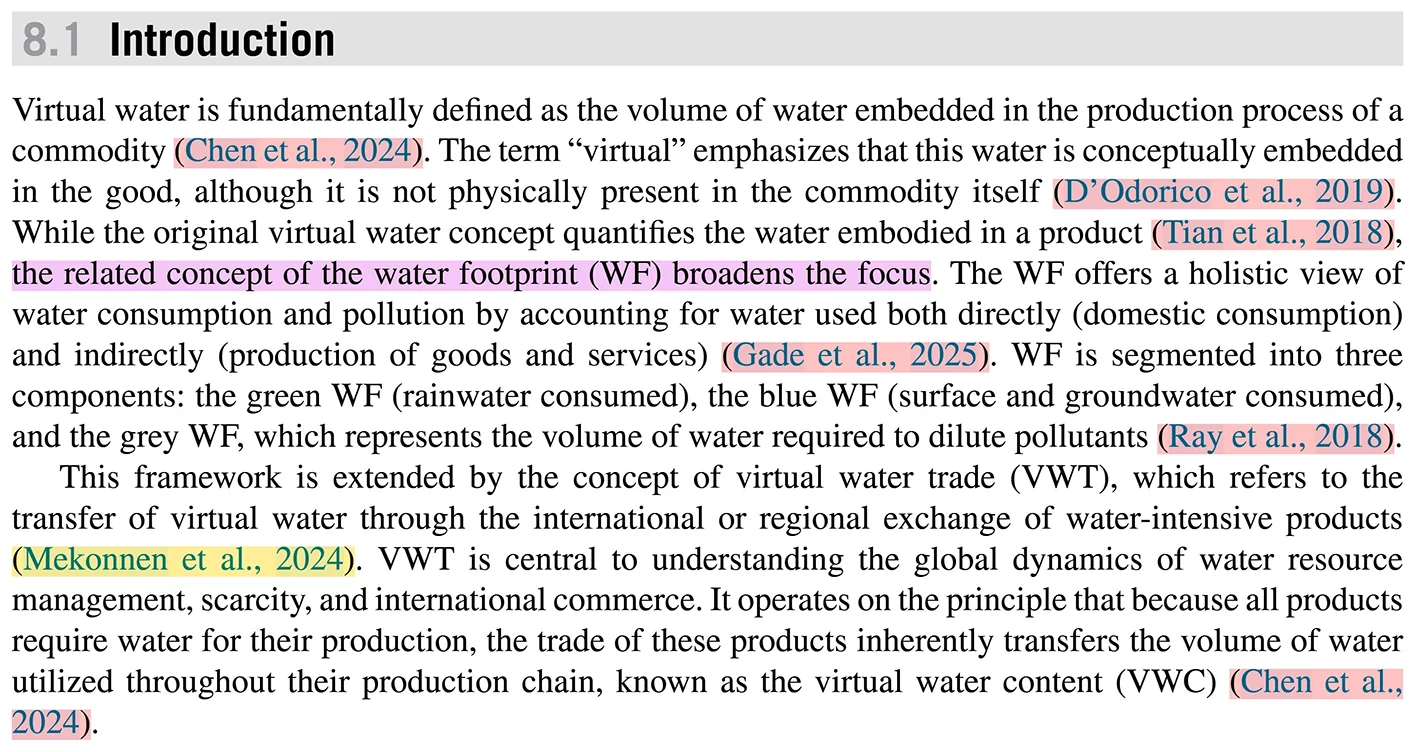
Illustrative example of semantically problematic citations (provided as an illustrative case rather than systematic empirical evidence). Red: citation of secondary sources instead of originators (credit to the wrong author); purple: missing citation of originator (obliteration by incorporation); yellow: citation of the correct author but an incorrect source. I conducted this empirical mini-analysis entirely manually, drawing exclusively on my domain expertise in water footprinting without utilizing AI assistance, to trace the original conceptual origins (e.g., J. A. Allan and A. Hoekstra).

As a reviewer, I am forced by the system into a deeply conflicted position: I am fully aware of what a rigorous peer-review process entails, yet a chronic lack of time forces me to make pragmatic compromises. To demonstrate what I mean in practical terms, Figure 1 shows an illustrative excerpt from a published paper. Since the specific paper is not important, I will not cite it here. I did not review this paper, so this is essentially an ex-post review of the paper after publication. I conducted this empirical mini-analysis entirely manually, drawing exclusively on my domain expertise in water footprinting without utilizing AI assistance, to trace the original conceptual origins (e.g., J. A. Allan and A. Hoekstra). The text shown contains 7 citations. The first sentence cites Chen et al. (2024). The problem is that they are neither the originators of the term “virtual water” nor the authors of its definition. The term and definition of virtual water were introduced by John Antony Allan back in the 1990s. Similarly, as early as the 1990s, J. A. Allan stated that virtual water is water that is not “physically present in the commodity” and that “the virtual water concept quantifies the water embodied in a product.” Therefore, the citations of D'Odorico et al. (2019) and Tian et al. (2018) are also unfounded. Conversely, the third sentence completely omits a citation of the originator of the water footprint concept, A. Hoekstra. Furthermore, the fourth sentence might lead an uninformed reader to believe that Gade et al. (2025) are the originators of the concept, but everything mentioned in the fourth sentence was published by A. Hoekstra between 2002 and 2008. The same applies to the content of the fifth sentence describing “water footprint colors”; again, this is not something invented by Ray et al. (2018), but was published by A. Hoekstra many years earlier. The same can be said about the second paragraph. Although M. Mekonnen belongs to a group of scientists who intensively study the water footprint and virtual water, the term “virtual water trade” has been in use since the 1990s and did not originate in 2024. Similarly, the term “virtual water content” does not date from 2024 but was used, for example, as early as the introduction of the water footprint in 2002.

## The limitations of conventional generative AI

I am able to conduct this empirical mini-analysis without consulting external sources, given that this is my primary field of research. Neither I, nor likely any other reviewer, possesses such detailed knowledge across all the topics cited in a single article today. Although no broad conclusions can be drawn from this brief analysis, it illustrates the challenges faced by the average reviewer. One potential way to address this problem is the integration of artificial intelligence. Modern large language models (LLMs) with web-browsing capabilities already demonstrate an impressive ability to analyze specific passages and verify, in isolation, whether a selected citation semantically aligns with the source statement. In preparing this manuscript, I evaluated Google's Gemini model on several test cases, and the preliminary results were highly promising. However, this approach does not constitute a systemic solution. The time required to input individual prompts was high, and when I submitted a request to assess citations across the entire manuscript, the model failed due to context length constraints and prompt refusal.

While this type of partial, *ad-hoc* assistance eases the reviewer's workload, in practice it encounters the rigid limitations of standard chat architecture. The first major barrier is academic paywalls; without integration into institutional library systems, standard conversational models cannot penetrate the locked databases of major publishers where the majority of full-text articles are located. Another limitation involves the timeout constraints of conventional online interfaces and the capacity of the context window, both of which prevent the simultaneous, deep parallel processing of dozens of multi-page studies.

Last but not least, there remains a persistent risk of well-documented contextual hallucinations, wherein a model generates fictitious information for the sake of text fluency that appears entirely legitimate at first glance (Alkaissi et al., 2023; Chauhan, 2024). To prevent these probabilistic models from overwhelming reviewers with false positives in highly specialized disciplines, where complex theoretical bridging or abstracted paraphrasing might be misflagged as semantic mismatches, semantic auditing agents should employ a calibrated confidence-scoring workflow. Rather than generating binary flags, the AI-driven semantic audit should calculate a semantic proximity score. Because accountability for the review report rests entirely with the human evaluator, the reviewer must retain full discretion over whether to manually inspect only citations falling below a predefined probability threshold. This probability-based routing ensures that reviewers focus exclusively on high-risk discrepancies, preventing ‘auditing the AI's audit' from becoming a new cognitive burden. Given these technical limitations, an AI-driven semantic audit can only serve as a supportive decision-making tool. Furthermore, as AI systems lack legal personhood and liability, accountability for audit accuracy rests entirely with the human user. Consequently, reviewers must always independently verify the generated findings.

Another perspective is worth mentioning. Using publicly accessible AI tools for peer review breaches authors' copyright and is discouraged or forbidden by most major publishers. Current conversational AI offers useful micro-support in case of doubts about specific sources, but for a comprehensive and systematic audit of an entire manuscript, it remains—due to the necessity of continuous human guidance, technological and legal restrictions—a tool of limited utility.

## AI-supported semantic auditing

It is at this critical point that the deployment of specialized autonomous AI agents (Agentic AI) emerges as a huge advance compared to current practice. While major scientific publishers, recognizing the unsustainability of the current situation, already provide reviewers with basic technical literature screening, this process remains largely mechanical and superficial. Existing publisher algorithms primarily verify whether a cited work exists, possesses a valid DOI, and is indexable in digital repositories. However, this level of control completely bypasses the core of the problem, as it fails to detect whether an author has distorted the source's actual conclusions or misused it as irrelevant filler. In contrast, true autonomous AI agents do not merely register a text's existence; they conduct a deep semantic audit, comparing the semantic meaning of a manuscript statement with the actual content of the source article on a sentence-by-sentence basis (Rensburg and Janse, 2025; Shi et al., 2026).

In the previous text, I only outlined a number of possible issues associated with the use of AI agents and the outputs they produce (legal liability, who will operate such an agent, how the risks of errors will be addressed, technical limitations, etc.). These issues will undoubtedly need to be discussed in much more detail than is possible in this paper. Given the goals of such a tool, publishers should play a central role in this discussion, since the work of a reviewer is voluntary, time-consuming (LeBlanc et al., 2023), economically highly valuable (Aczel et al., 2021) and unpaid. Such a tool represents a shift from mere “existence checking” to “meaning analysis” and may serve to transform peer review from an administrative ritual back into rigorous scientific verification.

## The missing link: mandatory citation reports

However, this technological tool encounters a fundamental epistemological limitation: neither a human reviewer nor the most advanced artificial intelligence can peer into the minds of authors and decipher their true motivations for placing a specific reference at a particular point in the text. An elegant and highly functional systemic solution to this challenge would therefore be for publishers to introduce a mandatory structured “citation report” alongside the manuscript submission. Within this matrix, authors would be required to state the line number, the exact source, and explicitly formulate the reason and context of the reference in one or two sentences for each individual citation.

A citation report would fully operationalize within publishing practice the classifications of the functional citation intent that computational linguistics and scientometrics have long defined theoretically (Teufel et al., 2006; Ihsan and Qadir, 2019). An automated semantic audit could then function with high accuracy, as it would not only compare the text with the abstract content of the source, but directly with the author's declared intention. Perhaps more importantly, this requirement would act as a powerful preventive filter against bibliometric inflation. Requiring authors to justify each individual reference would encourage self-regulation and the removal of superfluous citations prior to submission, thereby substantially easing the burden on the peer-review system.

A citation report should provide sufficient information to assess the relevance of the citation and to determine whether the citation supports or does not support the author's claims, or whether it is completely irrelevant (Martella et al., 2021). However, its specific form will have to be defined by individual publishers in collaboration with experts on the citation typing ontology and with regard to the requirements that individual editorial boards impose on manuscripts. Particularly in the case of publishers who apply the commendable principle of “Your Paper Your Way” (Davies, 2011), the precise localization of the citation within the text will be important. Furthermore, the citation report should specify the rhetorical function of the citation—that is, whether the citation serves to introduce, relate, utilize, explain, or compare (Dontcheva-Navratilova, 2016); and the role of the cited content, that is, what type of content from the cited work the author is incorporating, i.e., whether the citation refers to a method, data, results, or something else (Budi and Yaniasih, 2023). The citation report may also define the sentiment of the citation—that is, whether the citation is positive, negative, or neutral (Yousif et al., 2019) or any other connections between the citing and cited sources.

## Governance and implementation challenges

However, the proposed concept of a citation report also has its drawbacks; in particular, potential implementation obstacles must be considered. The extensive nature of the citation type ontology has already been mentioned. I therefore consider it appropriate for experts in citation type ontology and the publication process to define recommendations for the citation report. The lack of clear recommendations could lead to vague formulations of requirements for the citation report. Vaguely defined requirements for the citation report, in turn, could lead to overly formal formulations in the citation report and, in extreme cases, even make it impossible to audit citations. Without clear guidelines, non-native speakers may feel at a disadvantage, as they may not be able to distinguish subtle differences in meaning among the terms used in citation ontology, etc.

The aforementioned legal restrictions and paywall barriers could be effectively overcome if autonomous AI agents were implemented and operated directly at the publisher level. This publisher-driven approach rests on two core premises. First, publishers possess the technical infrastructure and institutional leverage required to safely operate these AI tools and access cross-publisher full texts. Second, because publishers directly benefit from the unpaid labor of reviewers, they have a responsibility to equip them with the best possible analytical resources. This would naturally extend current editorial practices, where publishers already supply reviewers with automated plagiarism reports (e.g., iThenticate) and basic reference validation. Furthermore, keeping AI agents under publishers' governance allows for the implementation of standardized safeguards against AI hallucination, false positives, false negatives, and the misinterpretation of specific disciplinary contexts. Ultimately, the responsibility for evaluating any AI-generated semantic discrepancies must remain firmly with the human reviewer.

Beyond technical error management, publisher governance of semantic AI is fundamentally an epistemological necessity. As highlighted by Biele and Kowalski (Biele and Kowalski, 2026), the integration of autonomous systems into peer review triggers a broader epistemic shift in science communication, redistributing evaluative authority among authors, reviewers, and algorithmic agents. Maintaining publisher-level oversight ensures that humans remain in control of the epistemological boundaries of science rather than delegating the validation of consensus entirely to automated systems.

Furthermore, to prevent this transition from creating a multi-tiered publishing ecosystem, where wealthy publishers deploy proprietary auditing tools while society-run or diamond open-access journals are left behind, the scientific community could advocate for shared, open-source infrastructure. Cooperative consortiums, analogous to Crossref or the Public Knowledge Project (PKP), could democratize access to semantic auditing tools, ensuring that citation integrity does not become a paywalled luxury.

The citation report will also place a greater burden on authors. Although I am an author of several publications myself, my perspective as a reviewer prevails. It is much easier for an author to justify the relevance of an individual citation than it is for any citation audit to do so. From my own experience, I can say that modern bibliographic management tools (e.g., Zotero or Mendeley) have made it so easy for authors to work with citations that it makes no difference whether a manuscript contains twenty or one hundred fifty cited sources. If a manuscript is rejected by one journal, I can reformat the citations to another journal's style in a matter of seconds.

Furthermore, addressing the burden on authors of citation reports is equally important. If authors feel stressed by generating justifications for dozens of references, they may resort to formulaic, boilerplate responses. And ironically, they can use generative AI to fabricate generic rationalizations. To mitigate this burden while avoiding the trap of rigid, ‘check-the-box' tagging, citation reporting should be integrated into the drafting phase via bibliographic management tools. Rather than restricting authors to predefined, static dropdown categories which risks encouraging superficial selection, such tools should prompt authors at the moment of reference insertion for a brief, open-text sentence stating the specific function of the source. Capturing authorial intent contextually during writing preserves genuine nuance, drastically reduces submission-stage administrative burden, and prevents formulaic *post-hoc* rationalization.

During the peer review of this article, the question was raised regarding the proportional application of the citation report (for example, only for selected types of citations, highly cited claims, review articles, or manuscripts flagged as high-risk). This is undoubtedly a highly relevant issue that publishers and the scientific community will have to address. As suggested during the peer review process, a phased rollout could effectively mitigate these initial implementation barriers. Publishers could introduce citation reports proportionally; for instance, piloting them strictly for high-risk manuscripts, review articles, or claims central to the manuscript's core argument. While my ultimate vision is that the citation report should comprehensively cover all citations, as they function as a primary scientific currency (Cronin, 1984), applying this tool selectively in the early stages represents a highly pragmatic, risk-averse pathway to adoption.

As an illustrative example of a minimalist citation report, I prepared a citation report for this article (Table 1). I spent only about 2 h preparing this report. Table 1 presents a foundational, minimalist version of a citation report as an initial model for discussion. Depending on their specific editorial needs, individual publishers could expand this framework to include additional fields, such as the manuscript line number, citation function, claim supported, or a verification note. When standardizing these requirements, publishers must strike a delicate balance between the analytical depth of the report and the administrative burden placed on authors. Authors should not be unduly overwhelmed, as the objective is to verify claims, not to compel them to produce a rigorous scientometric study in citation ontology. Conversely, the requirements must be stringent enough to prevent vague, formulaic justifications that would preclude an effective semantic audit and reduce the citation report to a mere bureaucratic formality.

**Table 1:** Minimal illustrative example of a citation report for this article.

Cited source	Localization	Author-declared reason
1	Introduction; paragraph 1	Foundational reference introducing the sociological role and ethical function of citations.
1	Impact on the review process and possible solutions; paragraph 2	Seminal work defining the theoretical concept of “obliteration by incorporation.”
2	Introduction; paragraph 2	Provides a historical overview of commercial paper mills and their evolution into sophisticated digital platforms.
3	Introduction; paragraph 2	Empirical study documenting the high rate of fabricated references (hallucinations) generated by large language models.
4	Introduction; paragraph 2	Empirical study documenting the high rate of fabricated references (hallucinations) generated by large language models.
5	Introduction; paragraph 3	One of the earliest empirical studies documenting citation inaccuracy.
6	Introduction; paragraph 3	Demonstrates how unverified information and mis-citations propagate and become entrenched within scholarly discourse.
7	Introduction; paragraph 4	Provides quantitative evidence of the “citation inflation” phenomenon.
8	Introduction; paragraph 4	Analyzes systematic shifts and inflationary trends in the structure of academic referencing.
9	Introduction; paragraph 4	Documents the longitudinal increase in the average number of references per academic article.
10	Impact on the review process and possible solutions; paragraph 1	Discusses the increasing time pressures of the peer-review process and authors' expectations for rapid publication.
11	Impact on the review process and possible solutions; paragraph 2	Case study emphasizing the importance of citing original authors rather than secondary literature.
12	Impact on the review process and possible solutions; paragraph 2	Key theoretical source expanding on the “obliteration by incorporation” phenomenon in scientometrics.
13	Impact on the review process and possible solutions; paragraph 6	Highlights the risks of artificial hallucinations when using conversational AI in scientific writing.
14	Impact on the review process and possible solutions; paragraph 6	Discusses the broader limitations and implications of generative AI in academic writing.
15	Impact on the review process and possible solutions; paragraph 7	Pioneering research proposing and evaluating AI-powered citation auditing.
16	Impact on the review process and possible solutions; paragraph 7	Pioneering research proposing and evaluating AI-powered citation auditing.
17	Impact on the review process and possible solutions; paragraph 8	Quantifies the extensive time commitment required by human reviewers during the peer-review process.
18	Impact on the review process and possible solutions; paragraph 8	Provides a financial estimate of the economic value of voluntary peer review.
19	Impact on the review process and possible solutions; paragraph 10	Describes frameworks for the classification and operationalization of citation intent and function.
20	Impact on the review process and possible solutions; paragraph 10	Describes frameworks for the classification and operationalization of citation intent and function.
21	Impact on the review process and possible solutions; paragraph 11	Demonstrates the practical application of classifying citations into supporting, non-supporting, or irrelevant categories.
22	Impact on the review process and possible solutions; paragraph 11	Editorial introducing the “Your Paper Your Way” submission principle, illustrating publisher-specific formatting flexibility.
23	Impact on the review process and possible solutions; paragraph 11	Key source defining the rhetorical functions of citations within academic discourse.
24	Impact on the review process and possible solutions; paragraph 11	Explores the semantic classification of citations based on their specific role.
25	Impact on the review process and possible solutions; paragraph 11	Investigates citation sentiment analysis (positive, negative, or neutral).
26	Impact on the review process and possible solutions; last paragraph	Classic text outlining the significance of citations as social currency within the scientific ecosystem (e.g. chapter Science as a Social System).

## Conclusion

In conclusion, the integration of autonomous AI agents into the reference verification process represents a timely response to growing challenges in academic integrity. When paired with editorial mechanisms such as authorial citation reports, deep semantic auditing may contribute to strengthening citation verifiability and reducing reviewer workload, provided these tools are implemented alongside appropriate human oversight, transparency, and governance safeguards. The objective of this integration is by no means to replace human judgment with automated algorithms. Rather, by delegating routine verification tasks to automated systems, human reviewers can reallocate time and cognitive resources toward the deep, contextual and truly creative and critical assessment of scientific ideas. Ultimately, an effective synergy between human expertise and machine precision can help ensure that the foundations of scientific knowledge remain solid, transparent, verifiable, and trustworthy.

## Data Availability

The original contributions presented in the study are included in the article/supplementary material, further inquiries can be directed to the corresponding author.
